# Dietary ω3 fatty acid exerts anti-allergic effect through the conversion to 17,18-epoxyeicosatetraenoic acid in the gut

**DOI:** 10.1038/srep09750

**Published:** 2015-06-11

**Authors:** Jun Kunisawa, Makoto Arita, Takahiro Hayasaka, Takashi Harada, Ryo Iwamoto, Risa Nagasawa, Shiori Shikata, Takahiro Nagatake, Hidehiko Suzuki, Eri Hashimoto, Yosuke Kurashima, Yuji Suzuki, Hiroyuki Arai, Mitsutoshi Setou, Hiroshi Kiyono

**Affiliations:** 1 Laboratory of Vaccine Materials, National Institute of Biomedical Innovation, Health and Nutrition, Osaka 567-0085, Japan; 2 Division of Mucosal Immunology, Department of Microbiology and Immunology, The Institute of Medical Science, The University of Tokyo, Tokyo 108-8639, Japan; 3 International Research and Development Center for Mucosal Vaccines, The Institute of Medical Science, The University of Tokyo, Tokyo, Japan; 4 Core Research for Evolutional Science and Technology (CREST), Japan Science and Technology Agency, Tokyo 102-0075, Japan; 5 Kobe University Graduate School of Medicine, Hyogo 650-0017, Japan; 6 Graduate School of Medicine, Graduate School of Pharmaceutical Sciences, and Graduate School of Dentistry, Osaka University, Osaka 565-0871, Japan; 7 Graduate School of Pharmaceutical Sciences, Osaka University, Osaka 565-0871, Japan; 8 Graduate School of Dentistry, Osaka University, Osaka 565-0871, Japan; 9 Laboratory for Metabolomics, RIKEN Center for Integrative Medical Sciences, Kanagawa 230-0045, Japan; 10 Graduate School of Medical Life Science, Yokohama City University, Kanagawa 230-0045, Japan; 11 Departments of Health Chemistry, Graduate School of Pharmaceutical Sciences, The University of Tokyo, Tokyo 113-0033, Japan; 12 Precursory Research for Embryonic Science and Technology (PRESTO), Japan Science and Technology Agency (JST), Tokyo 102-8666, Japan; 13 Department of Cell Biology and Anatomy, Hamamatsu University School of Medicine, Shizuoka 431-3192, Japan; 14 The Institute of Medical Science, The University of Tokyo, Tokyo 108-8639, Japan; 15 Department of Medical Genome Science, Graduate School of Frontier Science, The University of Tokyo, Chiba 277-8562, Japan; 16 Graduate School of Medicine, The University of Tokyo, Tokyo 113-0033, Japan

## Abstract

ω3 polyunsaturated fatty acids (PUFAs) have anti-allergic and anti-inflammatory properties, but the immune-metabolic progression from dietary oil remains to be investigated. Here we identified 17,18-epoxyeicostetraenoic acid (17,18-EpETE) as an anti-allergic metabolite generated in the gut from dietary ω3 α-linolenic acid (ALA). Biochemical and imaging mass spectrometry analyses revealed increased ALA and its metabolites, especially eicosapentaenoic acid (EPA), in the intestines of mice receiving ALA-rich linseed oil (Lin-mice). In murine food allergy model, the decreased incidence of allergic diarrhea in Lin-mice was due to impairment of mast cell degranulation without affecting allergen-specific serum IgE. Liquid chromatography–tandem mass spectrometry-based mediator lipidomics identified 17,18-EpETE as a major ω3 EPA-derived metabolite generated from dietary ALA in the gut, and 17,18-EpETE exhibits anti-allergic function when administered *in vivo*. These findings suggest that metabolizing dietary ω3 PUFAs generates 17,18-EpETE, which is an endogenous anti-allergic metabolite and potentially is a therapeutic target to control intestinal allergies.

Food allergies affect the quality of life of patients and their families; they may even cause severe or fatal reactions. Although the prevalence of food allergy has increased recently, current standards of care remain focused on the elimination of dietary allergens because available means of prevention and treatment are inadequate^1^. The immunologic mechanisms in the development of food allergy involve the disruption of oral tolerance, induction of Th2-type responses, allergen-specific IgE production, and mast cell (MC) activation^2^,^3^. These immune responses have been studied in several murine models of food allergy (including ours)^4^,^5^,^6^,^7^,^8^. Using egg white ovalbumin (OVA) as a model food allergen, we induce allergic diarrhea in mice accompanied by aberrant Th2-type responses, increased OVA-specific serum IgE, and MC infiltration and degranulation in the large intestine^4^; this type I intestinal allergy is therefore similar to that of human patients with egg food allergy. Our subsequent study shows that the development of intestinal allergy is mediated by sphingosine 1-phosphate by controlling the trafficking of pathogenic cells, such as Th2 cells and MCs^9^. Therefore, various host-derived factors (e.g., cytokines, antibodies, and lipid mediators) are likely involved in the development of intestinal allergy.

Immunologic crosstalk with nutritional components is also a critical determinant in the development of intestinal allergy and inflammation^10^,^11^; thus, deficient or inappropriate nutritional intake also increases the risk of infectious, allergic, and inflammatory diseases^12^. Among various nutritional factors, dietary oils (and especially fatty acid [FA] composition) are important immune regulators^13^,^14^. Dietary oils are typically composed of several long-chain FAs, including saturated (e.g., C16:0 palmitic acid and C18:0 stearic acid) and mono- (e.g., C18:1 oleic acid) or poly-unsaturated FAs (PUFAs; C18:2 linoleic acid [LA] and C18:3 α-linolenic acid [ALA]). Both LA and ALA are not generated by mammals and are thus obtained from the diet. LA, an ω6 PUFA, is converted into arachidonic acid (AA) by fatty acid elongase and subsequently into pro-inflammatory and pro-allergic lipid mediators^15^,^16^. In contrast, ALA, an ω3 PUFA, is converted in mammalian body to eicosapentaenoic acid (EPA) and docosahexaenoic acid (DHA), which are subsequently converted into anti-inflammatory and/or pro-resolving lipid mediators (such as resolvins and protectins)^13^,^17^. Because ω3 and ω6 PUFAs compete in metabolic pathways^18^, increased ω3 PUFA and decreased ω6 PUFA intake reduces the onset of aberrant murine and human immunologic conditions, including food allergy^19^,^20^,^21^; however, the effector lipid metabolites from the dietary oils to the regulation of food allergy are unknown.

In this study, we used matrix-assisted laser desorption ionization imaging mass spectrometry (MALDI-IMS)^22^ and liquid chromatography–tandem mass spectrometry (LC-MS/MS)-based lipidomics^13^ to investigate the metabolic progression of dietary oils in the regulation of intestinal immune system. Consequently, we identified ω3 EPA-derived metabolite derived from dietary ALA in the gut, which is a promising candidate for the prevention of intestinal allergy.

## Results

### Dietary ω3 ALA-enriched linseed oil prevents the development of allergic diarrhea by preventing effector phases of intestinal allergy

**Figure 1:**
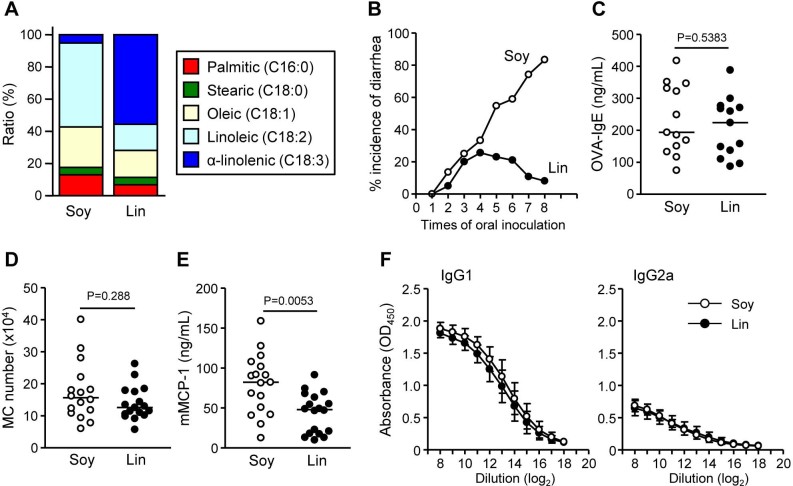
Decreased incidence of allergic diarrhea and MC degranulation in Lin-mice. (A) Fatty acid compositions of soybean (Soy) and linseed (Lin) oil. (B) After two months on diets containing 4% Soy or Lin oil, mice were systemically primed and orally challenged with OVA, after which the incidence of allergic diarrhea was measured (n = 40 from 8 experiments). (C–F) OVA-specific serum IgE production (C), MC counts in the large intestine (D), serum mMCP-1 production (E), and OVA-specific serum IgG1 and IgG2a production (F) were enumerated after the eighth oral challenge with OVA. Graphs show data from individual mice from 3 independent experiments, and bars indicate median values (C–E). The data represent the mean ± 1 SD (F, n = 12 from 2 independent experiments).

To examine whether ω3 PUFA-enriched diet affects intestinal allergy in egg allergy model, mice were maintained for 2 months on a diet of conventional amount (4%) of ω3 PUFA ALA-enriched linseed (Lin-mice) or control soybean (Soy-mice) oil (Figure 1A); we then induced OVA-specific allergic diarrhea in the mice. After several challenges with oral OVA, most Soy-mice exhibited allergic diarrhea whereas far fewer Lin-mice showed this symptom (Figure 1B). This allergic diarrhea in Soy-mice was associated with the induction of OVA-specific serum IgE, MC accumulation in the large intestine, and production of serum murine mast cell protease 1 (mMCP1; a marker of intestinal MC degranulation) (Figures 1C–E). Although fewer Lin-mice had allergic diarrhea, they exhibited similar levels of OVA-specific IgE in serum and increased MCs in the large intestine (Figures 1C and 1D). In addition, Soy- and Lin-mice had comparable OVA-specific IgG responses (Figure 1F). Specifically, IgG1 was predominant over IgG2a (Figure 1F), suggesting that Lin-mice have an unaltered Th1/Th2 balance. Consistently, no change was noted in the IL-20p40 production between Soy- and Lin-mice (287 ± 107 and 245 ± 21 pg/mL in Soy- and Lin-mice, respectively). In contrast, among several allergic mediators (e.g., histamine, serotonin, platelet-activating factor, and eotaxin)^4^,^5^, Lin-mice had decreased serum mMCP-1 compared with Soy-mice in our experimental condition (Figure 1E); their decreased incidence of allergic diarrhea is likely due to the prevention of effector phase of intestinal allergy (e.g., MC degranulation) rather than sensitization phase.

### Dietary FAs affect the in vivo lipid composition

**Figure 2:**
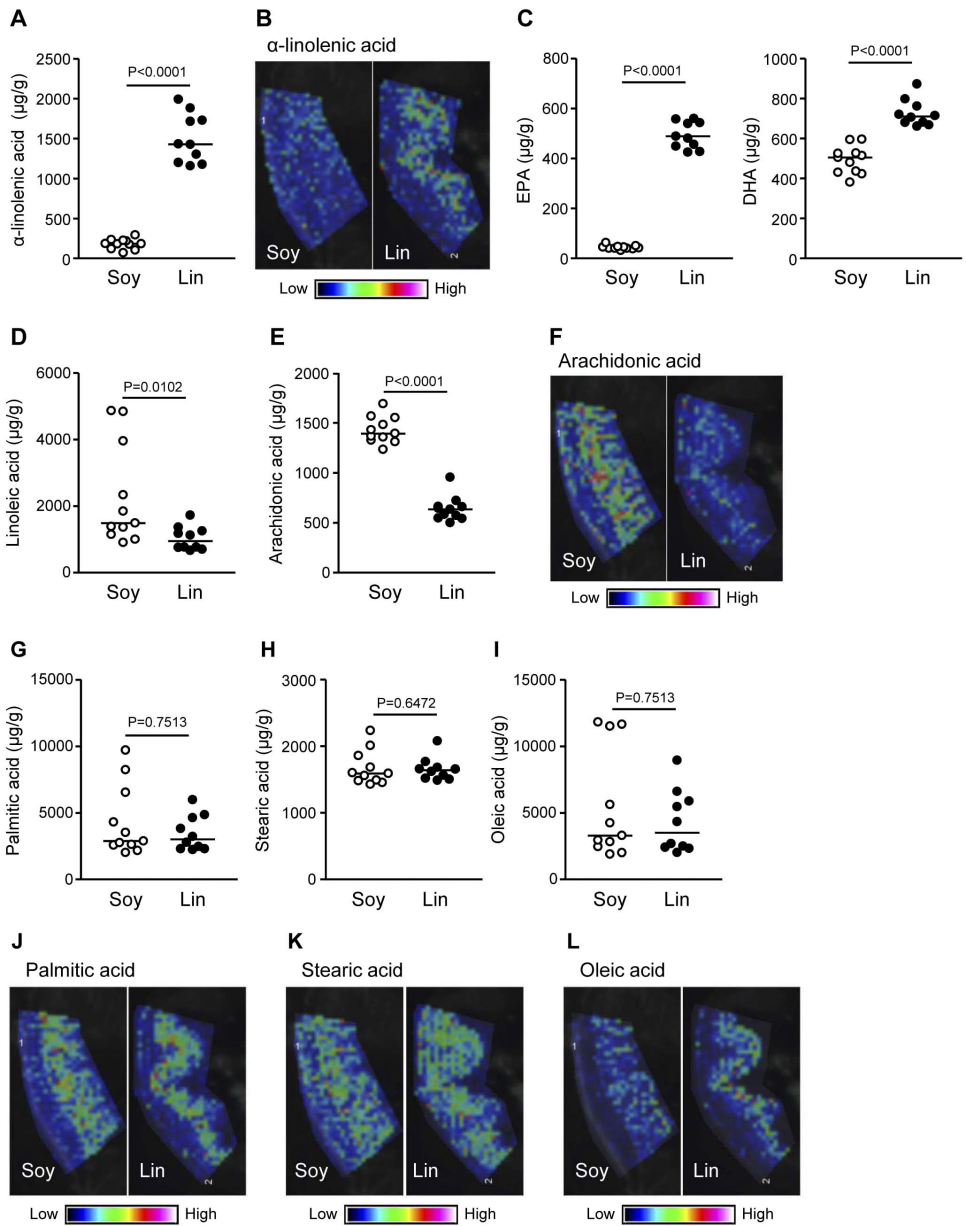
Fatty acid composition and distribution in the large intestines of mice receiving different dietary oils. After two months of maintaining mice on diets containing 4% soybean (Soy) or linseed (Lin) oil, large intestines were collected for measuring α-linolenic acid (A), EPA and DHA (C), linoleic (D), arachidonic (E), palmitic (G), stearic (H), and oleic (I) acids by gas chromatography or for the detection of α-linolenic (B), arachidonic (F), palmitic (J), stearic (K), and oleic (L) acids by MALDI-IMS. Graphs show data from individual mice, and bars indicate median values. MALDI-IMS images are representative from three independent experiments.

We then investigated whether dietary FA composition affects the lipid composition in the large intestine. First, we measured the amount of ALA in the large intestine because linseed oil contains abundant ALA (Figure 1A). As expected from the lipid composition of the dietary oils, the amount of ALA in the large intestine of Lin-mice was higher than that of Soy-mice (Figure 2A). We then used MALDI-IMS to visualize ALA distribution in the large intestine. Whereas Soy-mice were largely devoid of ALA-specific signal, Lin-mice had abundant ALA, especially in the villi region where many immune cells are present (Figure 2B). We next measured the ALA-derived metabolites, EPA and DHA, in the large intestine; compared with Soy-mice, Lin-mice had increased EPA and DHA (Figure 2C). Reciprocally, the large intestinal lamina propria of Lin-mice had less LA and its metabolite, AA, than did Soy-mice (Figures 2D–F).

**Figure 3:**
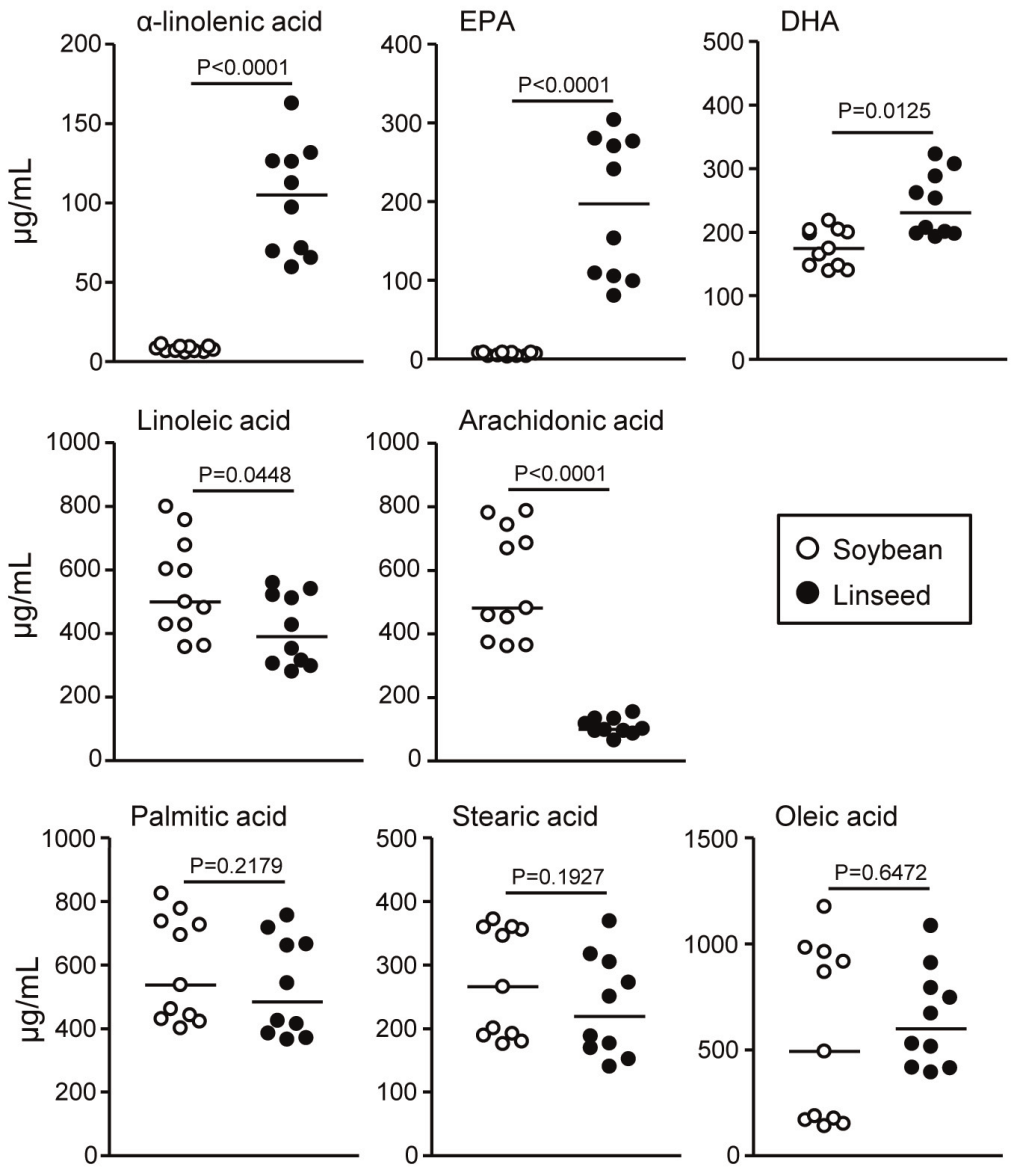
Fatty acid composition in the serum of mice receiving different dietary oils. After mice were maintained for two months on diets containing 4% soybean (open) or linseed (closed) oil, serum was collected for the measurement of fatty acids by gas chromatography. Graphs show data from individual mice from 2 individual experiments, and bars indicate median values.

In contrast, the amount and distribution of non-essential palmitic, stearic, and oleic acids were comparable between Soy- and Lin-mice (Figures 2G–L). In addition, the levels of these FAs in the large intestine were consistently similar to those in the serum. Indeed, the serum concentrations of ω3 PUFAs (e.g., ALA, EPA, and DHA) were higher and those of ω6 PUFAs (e.g., LA and AA) were lower in Lin-mice than in Soy-mice without significant differences in the levels of non-essential FAs between the mice (Figure 3).

### 17,18-EpETE is specifically produced in the large intestine of allergy-inhibited mice

**Figure 4:**
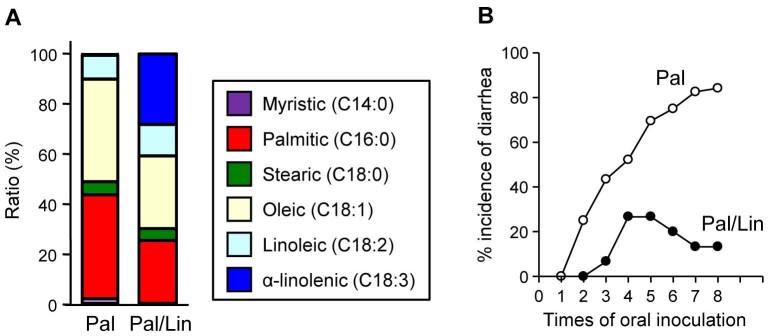
Increased ALA is sufficient to decrease the incidence of allergic diarrhea. (A) Fatty acid composition in palm (Pal) and equally mixed palm and linseed (Pal/Lin) oils. (B) After two months on diets containing 4% Pal or Pal/Lin oils, mice were used in OVA-induced intestinal allergy model, and the incidence of allergic diarrhea was measured (n = 30 from 6 individual experiments).

Because Lin-mice simultaneously exhibited increases in ω3 PUFAs and decreases in ω6 PUFAs, whether one factor or both contributes to the protection against intestinal allergy was unclear. To address this issue, we used palm oil, which uniquely contains very little ALA, but its proportion of LA is nearly the same as for linseed oil (Figure 4A). Therefore, when equal amounts of palm and linseed oils are mixed, the ALA content is increased with little effect on the LA content (Figure 4A). We maintained mice on a 4% palm oil (Pal-mice) or mixed oil (2% palm and 2% linseed oil; Pal/Lin-mice) diet and then induced OVA-specific allergic diarrhea. The incidence of allergic diarrhea was noted in Pal-mice but markedly decreased in Pal/Lin-mice (Figure 4B). Therefore, although we cannot completely rule out the effect of decreased ratio of LA in Lin-mice on the reduction of allergic diarrhea incidence, increased ALA is likely sufficient to decrease the incidence of allergic diarrhea.

**Figure 5:**
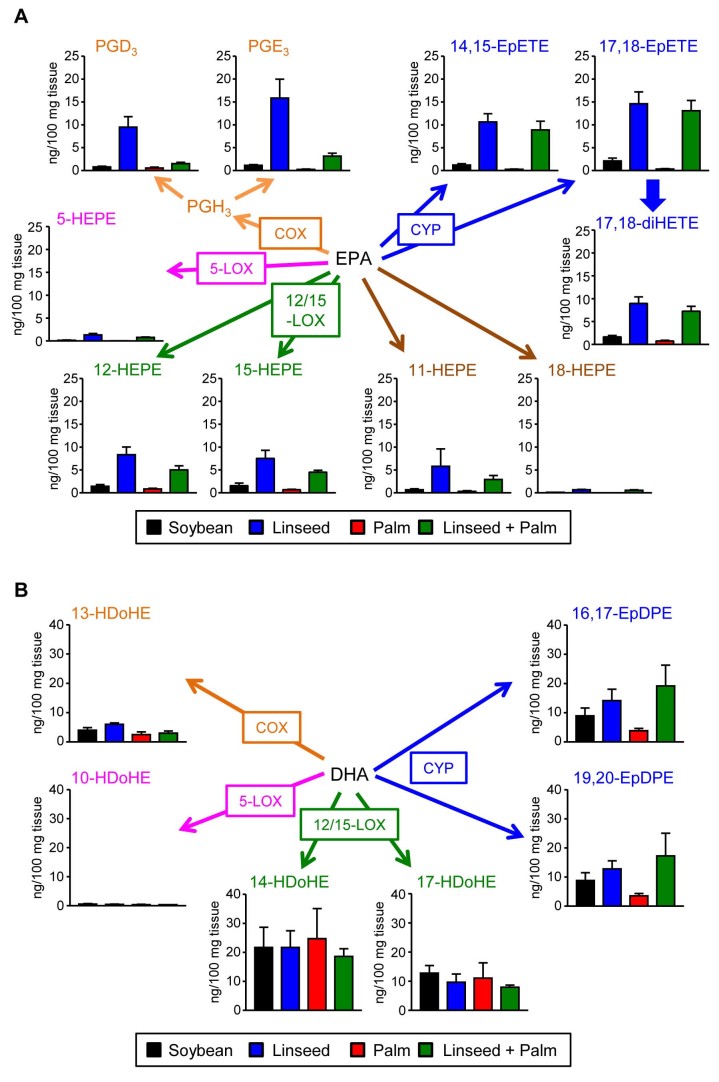
Different levels of EPA-derived fatty acid metabolites in the large intestines of mice receiving different dietary oils. Mice were maintained for two months on diets containing 4% soybean (black), linseed (blue), palm (red), or linseed + palm (green) oils, large intestines were isolated to measure EPA- (A) or DHA- (B) derived fatty acid metabolites by LC-MS/MS. Data are given as means ± 1 SD (n = 8 from 2 individual experiments).

Given these results, we next investigated FA-derived mediator profiles in the gut. For the comprehensive and quantitative measurement of the FA-derived lipid mediators generated by several enzymes (e.g., cyclooxygenase [COX], 5-lipoxygenase [5-LOX], 12/15-LOX, and cytochrome P450 [CYP]), we performed unbiased target lipidomics by using LC-MS/MS. Our analyses identified several hydroxylated products that were increased in the large intestine of Lin-mice. Of note, many of these increased lipid mediators were derived from EPA but not DHA (Figures 5A and 5B). Among them, 17,18-epoxyeicostetraenoic acid (17,18-EpETE) was one of the most prominent metabolites increased in both Lin- and Pal/Lin-mice but not in Soy- and Pal-mice (Figure 5A).

### Exogenous 17,18-EpETE inhibits the development of intestinal allergy

**Figure 6:**
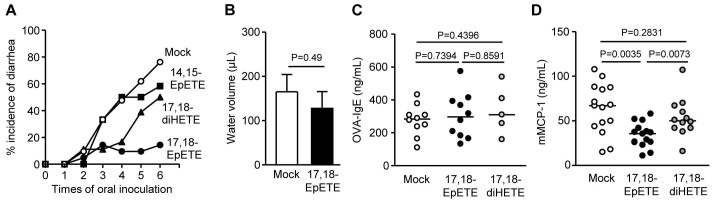
17,18-EpETE prevents the development of allergic diarrhea by impairing MC degranulation. (A) Mice were injected i.p. without (mock) or with 100 ng 17,18-EpETE, 14,15-EpETE, or 17,18-diHETE 30 min before systemic priming and oral challenge with OVA, after which the incidence of allergic diarrhea was measured (n = 16 per each group). (B) Mice were injected i.p. without (mock) or with 100 ng 17,18-EpETE at 24 and 1 hr before oral inoculation of 25 μg cholera toxin. Fifteen hours after oral administration of cholera toxin, water volume in the intestinal lumen was measured. The data represent the mean ± 1 SD (n = 4). (C, D) One day after the eighth oral challenge with OVA, serum was collected for the measurement of OVA-specific IgE (C) and mMCP-1 (D) levels. Graphs show data from individual mice from 2 individual experiments, and bars indicate median values.

To determine if 17,18-EpETE is sufficient to decrease the incidence of allergic diarrhea, synthetic 17,18-EpETE was intraperitoneally injected. As with Lin-mice, injection of synthetic 17,18-EpETE during the induction of intestinal allergy decreased the incidence of allergic diarrhea (Figure 6A), but not cholera toxin-induced diarrhea, an example of pathogenic toxin causing diarrhea (Figure 6B), suggesting that 17,18-EpETE specifically inhibits allergy-associated diarrhea. We also found that 14,15-EpETE, another CYP-mediated metabolite of EPA, was not effective to prevent allergic diarrhea (Fig. 6A), suggesting that epoxidation site in EPA is a decisive factor in the prevention of allergic diarrhea. In addition, like Lin-mice (Figure 1), treatment with 17,18-EpETE reduced mMCP-1 levels without affecting OVA-specific IgE levels (Figures 6C and 6D).

In the body, 17,18-EpETE can be rapidly hydrolyzed by epoxide hydrolase to form 17,18-dihydroxy-eicosa-5,8,11,14-tetraenoic acid (17,18-diHETE)^23^. Because, like 17,18-EpETE, 17,18-diHETE was increased in the large intestine of Lin- and Pal/Lin-mice compared with Soy- and Pal-mice (Figure 5A), we next investigated whether17,18-diHETE functions similarly, and found the 17,18-diHETE had little effect on the incidence of allergic diarrhea or production of mMCP-1 and OVA-specific IgE (Figures 6A–C). Therefore, EPA-derived 17,18-EpETE itself is likely an ω3 PUFA metabolite responsible for preventing intestinal allergy.

## Discussion

In this study, we investigated dietary FA matabolism in the intestine and its association with the development of intestinal allergy. In host tissues (e.g., intestine and serum), the FA composition of dietary oils directly reflects the levels of essential ω3 and ω6 PUFAs but not of non-essential FAs. Imaging analyses showed the lipid distribution in the large intestine, revealing that ALA- and LA-related lipid metabolites are present mainly in the lamina propria rather than muscle region of the large intestine. In general, FAs in dietary oils are present primarily as triacylglycerol and are digested by lipases into monoacylglycerol plus free FAs. After absorption by epithelial cells, these FAs are reconstituted to triacylglycerol, are incorporated into chylomicrons, and subsequently circulate through lymphatics and blood. Thus, after the absorption of ALA and LA in the intestine, they (and their metabolites) recirculate into the lamina propria of the large intestine; there they can affect immune cells.

EPA and DHA are categorized as ω3 PUFAs with similar functions and properties; however, our study in mice shows that the amount of ALA in dietary oils preferentially reflects the composition of EPA-derived metabolites with little influence on DHA. Similar findings were previously reported in human studies^24^. E-series resolvins derived from EPA are well known as anti-inflammatory mediators^13^,^17^; however, E-series resolvins and 18-HEPE, a precursor of E-series resolvins, were increased only slightly in Lin-mice in our experimental condition. Alternatively, 17,18-EpETE was produced abundantly in the large intestine of Lin-mice; notably, it actively inhibited intestinal allergy. 17,18-EpETE is generated by CYP from EPA, and it is known that lipid mediators generated by CYP regulate inflammatory, vascular, cardiac, and renal functions^25^,^26^,^27^. In addition, 17,18-EpETE and its bioactive metabolite 12-hydroxy-17,18-EpETE is increased in the peritoneal fluid of mice maintained on an EPA-enriched diet^28^. Therefore, our current study furthers the field of nutritional and lipid biology by demonstrating the pathway of CYP-mediated 17,18-epoxygenation of EPA to generate anti-allergic lipid metabolite, 17,18-EpETE.

Among CYPs, CYP1A, CYP2C, and CYP2J subfamily members can introduce a *cis*-epoxide at EPA to generate 17,18-EpETE^29^,^30^,^31^. In addition, CYP has polymorphisms^32^. Therefore, the subfamily expression and polymorphisms of CYP may explain the controversy regarding the beneficial effects of ω3 PUFAs on inflammation and allergy in humans^21^,^33^. Furthermore, the anti-allergy activity of 17,18-EpETE is abolished by its further conversion into 17,18-diHETE. Indeed, the inhibition of epoxide hydrolase (a key enzyme converting 17,18-EpETE into 17,18-diHETE) ameliorates inflammatory responses^34^. Therefore, in addition to the substrates of lipid mediators (e.g., EPA), enzyme expression in the generation and conversion of 17,18-EpETE likely determines the effects of ω3 PUFAs in the control of intestinal allergy.

IgE-mediated MC degranulation is strongly associated with the pathophysiology of allergic reactions, including food allergy. Our current study showed the impaired MC degranulation and thus decreased mMCP-1 accompanied the decreased incidence of allergic diarrhea. Although the pathologic function of mMCP-1 in the development of intestinal allergy remains to be investigated, mMCP-1 increases intestinal permeability in a parasite infection model^35^. Therefore, the reduction of mMCP-1 in Lin-mice or 17,18-EpETE-treated mice likely prevents allergic diarrhea by controlling intestinal permeability. In contrast to the *in vivo* results, our preliminary study indicated that 17,18-EpETE only partially inhibited IgE-mediated MC degranulation *in vitro*; this finding suggests alternative inhibitory pathways. There are several possibilities to explain the difference between the *in vivo* and *in vitro* results; the heterogeneity of MCs among tissue environments^36^, change in or decrease of content of allergic mediators with little effect on MC degranulation, inhibition of IgE-independent MC degranulation (e.g., eosinophil major basic protein, vasoactive intestinal peptide, and complement C5a)^37^,^38^,^39^, augmentation of signaling through inhibitory molecules (e.g., PIR-B and allergin I)^40^,^41^, and indirect effects through other cells (e.g., stromal cells)^42^.

In summary, we demonstrate the metabolic progression from dietary oils, particularly ω3 ALA, to the generation of the anti-allergy lipid mediator 17,18-EpETE. 17,18-EpETE is an endogenous ω3 ALA metabolite and efficiently decreases allergic diarrhea; it is therefore a promising candidate for a safe and effective anti-allergic compound to prevent intestinal allergy.

## Methods

### Mice

Female Balb/c mice (6 weeks old) were purchased from Japan Clea (Tokyo, Japan) and maintained for 2 months on diets composed of chemically defined materials with 4% each dietary oil (Oriental Yeast, Tokyo, Japan)^43^. All animals were maintained in the experimental animal facilities of the University of Tokyo and National Institute of Biomedical Innovation. The experiments were approved by the Animal Care and Use Committees of both institutes and were conducted in accordance with their guidelines.

### Induction of diarrhea

OVA-specific allergic diarrhea was induced as previously described^4^,^9^. Briefly, mice were primed by s.c. injection of 1 mg OVA (Sigma-Aldrich, St. Louis, MO) in complete Freund's adjuvant (Difco Laboratories, Detroit, MI). One week after systemic priming, mice were challenged orally with 50 mg OVA and continued to be challenged 3 times each week. We assessed allergic diarrhea 30 to 60 min after oral inoculation with OVA.

Cholera diarrhea was induced by oral administration of 25 μg cholera toxin (List Biological Laboratories, Campbell, CA)^44^. Fifteen hours later, we examined the water volume in the intestinal lumen.

### Cell isolation

Cells were isolated from the large intestine as previously described^44^,^45^. Briefly, intestines were opened longitudinally, washed with RPMI-1640, cut into 2-cm pieces, and stirred for 20 min at 37°C in RPMI-1640 containing 0.5 mM EDTA and 2% FCS to remove epithelial cells and intraepithelial lymphocytes. The tissues were then stirred three times (20 min each) in 1.6 mg/ml collagenase (Wako, Osaka, Japan).

### Flow cytometry

Cells were pre-incubated with 10 μg/mL anti-CD16/32 antibody (Biolegend, San Diego, CA) and then stained with an antibody specific to c-kit (BD Biosciences, San Diego, CA) and FcεR1α (eBioscience, San Diego, CA) for 30 min at 4°C. We used FSC-H and FSC-A discrimination to exclude doublet cells and Viaprobe Cell-viability Solution (BD Biosciences) to discriminate dead and living cells. Flow-cytometric analysis was performed by using a FACSCantoII (BD Biosciences).

### Measurement of mMCP-1, OVA-specific IgE, and IgG by ELISA

OVA-specific IgE and mMCP-1 production in serum was measured by using DS Mouse IgE (OVA) ELISA kit (DS Pharma Biomedical Co., Osaka, Japan) and Mouse MCP-1 ELISA kit (eBioscience), according to the manufacturers' protocols. OVA-specific IgG1 and IgG2a were measured as previously reported^44^. Briefly, plates were coated with 1 mg/mL OVA in PBS; this was followed by blocking for 1 hr at room temperature with 200 μL PBS containing 1% (w/v) BSA per well. After extensive washing of the plates with PBS containing 0.05% Tween 20, serial serum dilutions were added for incubation overnight at 4°C. Samples were then incubated for 1 hr at room temperature with optimally diluted HRP-conjugated goat anti-mouse IgG1 or IgG2a (SouthernBiotech, Birmingham, AL). After sample washing, the color reaction was developed at room temperature by using 3,3′,5,5′-tetramethylbenzidine (KPL, Baltimore, MD) and terminated by adding 0.5 M HCl. We measured the color reaction as the absorbance at 450 nm.

### Gas chromatography

We extracted lipids from serum and large intestine by using chloroform–methanol and chloroform solutions. The specimens were dried in nitrogen gas and dissolved in 0.4 M potassium methoxide in methanol and 14% boron trifluoride in methanol. The FA concentrations in the solutions were measured by using gas chromatography (model GF 17A; Shimazu, Kyoto, Japan) at SRL Inc. (Tokyo, Japan).

### MALDI-IMS

Large intestines within 2 cm from the ileal end were isolated. After the intestinal lumen was washed with PBS, the mesenteries were removed, and the intestines were cut into 2-cm lengths. The intestines were frozen in 2% carboxymethylcellulose (Wako, Osaka, Japan) dissolved in ultra-pure water. Before sectioning, the frozen samples were kept for 30 min at −20°C. The 10-μm sections were thaw-mounted onto an indium–tin–oxide-coated glass slide (Bruker Daltonics, Bremen, Germany) and dried at room temperature. The sections were placed in a polycarbonate tube and stored at −20°C until IMS analysis.

We performed the matrix deposition of 9-aminoacridine (Merck Schuchardt, Hohenbrunn, Germany) onto a slide in a sublimation apparatus (Shimadzu, Kyoto, Japan). IMS was performed with a MALDI TOF/TOF-type instrument, the Ultraflex II (Bruker Daltonics Bremen, Germany), which was equipped with a 355-nm Nd/YAG laser with a repetition rate of 200 Hz. All pixel sizes of imaging were 100 μm. The MS parameters were set in the range of *m/z* (200–400) in negative-ion mode. Automatic acquisition of the mass spectra and reconstruction of the ion images were performed by using FlexImaging software (Bruker Daltonics), which normalized all mass spectra based on total ion current.

### Detection of FAs and their metabolites in the large intestine

LC-MS/MS-based lipidomics was performed to measure the amounts of lipid mediators as previously reported^13^. Briefly, lipids were collected by solid-phase extraction using Sep-Pak C18 cartridge (Waters) with a deuterium-labeled internal standard (AA-d8, 15-HETE-d8, LTB4-d4, and PGE2-d4). We used a triple quadrupole linear ion trap mass spectrometer (QTRAP5500; AB SCIEX) equipped with a 1.7 μm, 1.0 × 150 mm Acquity UPLC™ BEH C18 column (Waters). The MS/MS analyses were performed in negative ion mode, and FA metabolites were identified and quantified by multiple reaction monitoring.

### Statistics

Results were compared by non-parametric Mann–Whitney's *U*, two-tailed unpaired *t*, and One-way ANOVA tests (GraphPad Software, San Diego, CA).
